# Correction of inter‐scan motion artifacts in quantitative R1 mapping by accounting for receive coil sensitivity effects

**DOI:** 10.1002/mrm.26058

**Published:** 2015-11-26

**Authors:** Daniel Papp, Martina F. Callaghan, Heiko Meyer, Craig Buckley, Nikolaus Weiskopf

**Affiliations:** ^1^ Wellcome Trust Centre for Neuroimaging, UCL Institute of NeurologyLondonUnited Kingdom; ^2^ SIEMENS Healthcare GmbHErlangerGermany; ^3^ SIEMENS Plc (Healthcare Division)CamberleyUnited Kingdom; ^4^ Department of NeurophysicsMax Planck Institute for Human Cognitive and Brain SciencesLeipzigGermany.

**Keywords:** quantitative MRI, R1 mapping, motion correction, receive sensitivity, VFA, MPM

## Abstract

**Purpose:**

Inter‐scan motion causes differential receive field modulation between scans, leading to errors when they are combined to quantify MRI parameters. We present a robust and efficient method that accounts for inter‐scan motion by removing this modulation before parameter quantification.

**Theory and Methods:**

Five participants moved between two high‐resolution structural scans acquired with different flip angles. Before each high‐resolution scan, the effective relative sensitivity of the receive head coil was estimated by combining two rapid low‐resolution scans acquired receiving on each of the body and head coils. All data were co‐registered and sensitivity variations were removed from the high‐resolution scans by division with the effective relative sensitivity. R1 maps with and without this correction were calculated and compared against reference maps unaffected by inter‐scan motion.

**Results:**

Even after coregistration, inter‐scan motion significantly biased the R1 maps, leading to spurious variation in R1 in brain tissue and deviations with respect to a no‐motion reference. The proposed correction scheme reduced the error to within the typical scan–rescan error observed in datasets unaffected by motion.

**Conclusion:**

Inter‐scan motion negatively impacts the accuracy and precision of R1 mapping. We present a validated correction method that accounts for position‐specific receive field modulation. Magn Reson Med 76:1478–1485, 2016. © 2015 The Authors. Magnetic Resonance in Medicine published by Wiley Periodicals, Inc. on behalf of International Society for Magnetic Resonance in Medicine. This is an open access article under the terms of the Creative Commons Attribution License, which permits use, distribution and reproduction in any medium, provided the original work is properly cited.

## INTRODUCTION

Participant motion is a significant source of artifacts in MRI. This motion can occur in two distinct forms: intrascan motion occurring within a scan, and inter‐scan motion occurring between two scans.

The majority of current MRI methods are qualitative single‐scan approaches, where diagnostic information is derived from image contrast, and different scans are evaluated separately or compared within qualitative frameworks. Such MRI methods are susceptible to intra‐scan motion that degrades the image quality of individual scans. Consequently, several methods, both prospective and retrospective, have been developed to address intrascan motion, most recently reviewed by Zaitsev et al 1.

Several quantitative imaging methods rely on combining data from multiple acquisitions from a single session. As such, they are not only susceptible to intra‐scan motion, but also to inter‐scan motion. For example, estimation of the longitudinal relaxation rate (R1) in the variable flip angle (VFA) framework 2, 3 combines data from at least two scans, and is therefore vulnerable to inter‐scan motion. Such estimation methods include DESPOT1/DESPOT2 4 and multi‐parameter mapping 5, 6 as used in this study.

To date, inter‐scan motion has not been addressed to the same extent as intra‐scan motion. One approach to correcting inter‐scan motion is to perform three‐dimensional (3D) affine co‐registration of the different scans. This re‐aligns the images to achieve spatial correspondence between scans 7. Navigators have also been used to monitor and correct for inter‐scan motion in a clinical framework 8. Intra‐scan motion has also been addressed by transforming it into inter‐scan motion, by splitting up long acquisitions into several shorter ones, and subsequently co‐registering them 9. It is important to note that all of these methods were developed for conventional, non‐quantitative MRI.

As multi‐channel radiofrequency (RF) receive head coils are routinely used in clinical practice and research, data acquired with such array coils show an additional signal intensity modulation corresponding to the overall receive sensitivity field of the coils. Inter‐scan motion within this receive sensitivity field changes the modulation pattern from scan to scan, because the distance and orientation of the head with respect to the coil elements changes.

In this work, we demonstrate the impact of inter‐scan motion on quantitative estimation of the longitudinal relaxation rate (R1) using a multiparameter mapping approach 5. We propose a correction method, based on removing the spatial signal intensity modulation caused by the receive field specific to each scan. We derive these receive sensitivity fields from two fast low‐resolution acquisitions, acquired with the RF body and head coils, before each high‐resolution scan. Our proposed method is in principle applicable to all quantitative methods that rely on combining data from more than one scan.

## THEORY

### Inter‐scan Motion

To describe the effects of inter‐scan motion, we introduce a coordinate system that is fixed to the brain (instead of the RF head coil). For a multi‐scan protocol, this is equivalent to inter‐scan motion correction by rigid body co‐registration, because anatomical features are effectively tracked and matched. The magnitude of the MRI signal in this coordinate system is:

(1) $$S_{A}\left(r\right)=C\left(r\right)\cdot S_{0,A}\left(r\right)$$

where r is the spatial position, S_A_(r) is the detected signal intensity of scan A, C(r) is the magnitude of the combined receive sensitivity field of the multichannel head coil, and S_0A_(r) is the unmodulated signal intensity of scan A, which is determined by the anatomy and the acquisition parameters.

If another scan is acquired after head movement then, after rigid body motion correction, the magnitude of the MR signal is:

(2) $$S_{RB,B}\left(r,r^{\prime }\right)=C\left(r\prime \right)\cdot S_{0,B}\left(r\right);r^{\prime }=r+\Delta r$$

where r is the spatial position, S_RB,B_(r,r′) is the detected signal intensity of scan B after rigid body motion correction to scan A, Δr is the difference in position between the two scans, C(r′) is the magnitude of the combined receive sensitivity field of the multichannel head coil at the new position r′, and S_0,B_(r) is the unmodulated signal intensity of scan B, again determined by the anatomy and the acquisition parameters, as illustrated in Figure 1a.

**Figure 1:**
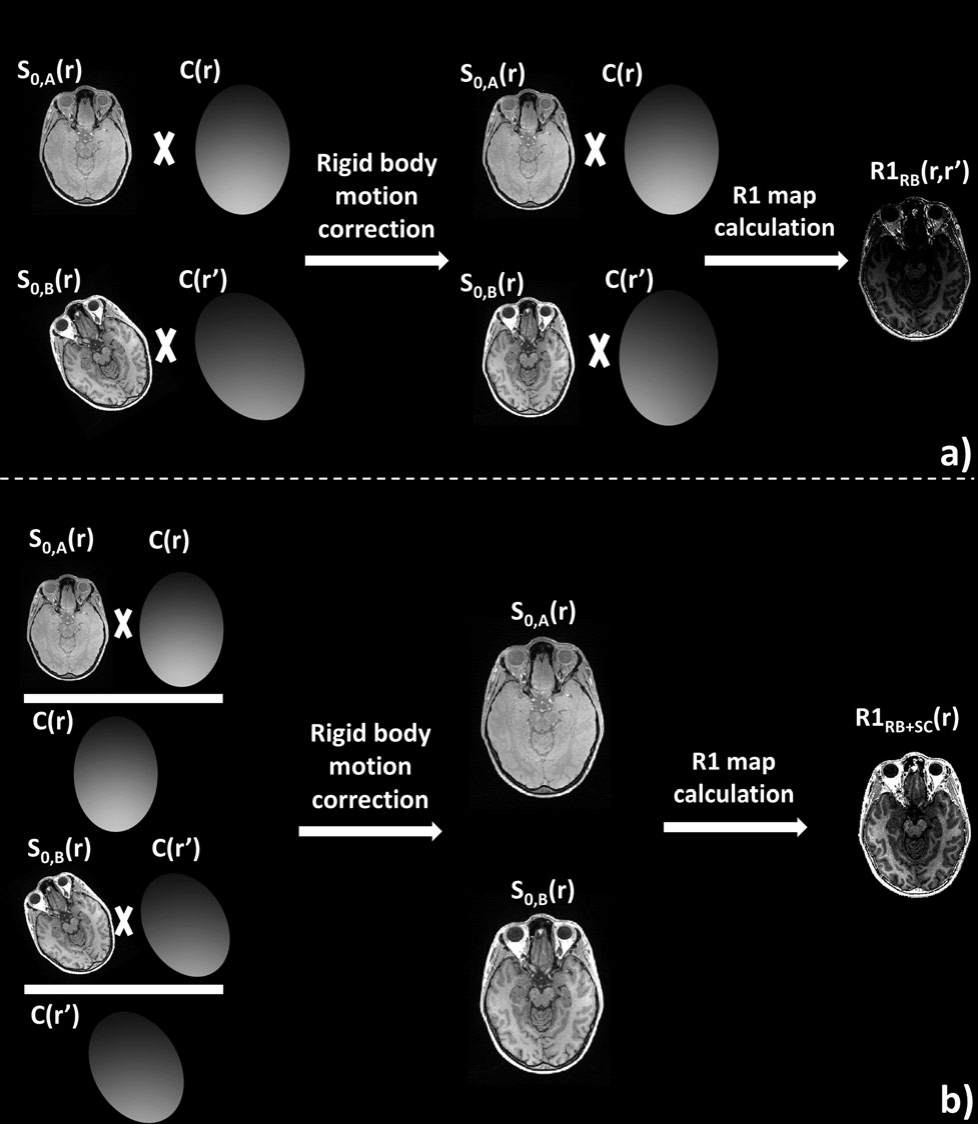
The effect of interscan motion is illustrated, using two simulated receive sensitivity fields and an exaggerated rotational motion. **a**: In the case of interscan motion, each scan is modulated by a different receive field. Rigid body motion correction does not correct for this effect, and data derived from these two scans will show a spatially varying bias due to the different receive fields. **b**: The proposed correction method removed the effect of the receive fields by means of division, removing the bias.

In Eqs. (1) and (2), S_0,A_(r) and S_0,B_(r) are driven only by the anatomy and the respective acquisition parameters and would be the ideal basis for quantification, e.g., of R1. However, in Eq. (2), the detected signal S_RB,B_(r,r′) is dependent on both r and r′ and is affected by a different receive field sensitivity, which leads to bias in the derived R1 map, as illustrated in Figure 1a.

Standard rigid body inter‐scan motion correction does not account for the different modulations due to the receive sensitivity field. If the sensitivity field C(r) was known, it could be removed from the signal by division. This is the basis of our proposed correction method.

### Correction of Receive‐Sensitivity‐Related Inter‐scan Motion Artifacts

We propose a method that incorporates a correction for motion‐related relative receive sensitivity variations in addition to performing rigid body realignment. To this end, we measure the receive sensitivity field C(r) before each scan. Over the spatial extent of the head, we assume the receive sensitivity of the body coil to be flat 10. If the same anatomy is imaged with the head coil and the body coil sequentially, using the same acquisition parameters and assuming no motion, then the ratio of these two scans ( β(r)) is the head coil receive sensitivity field , divided by a constant.

(3) $$S_{HC}\left(r\right)=C\left(r\right)\cdot S_{0}\left(r\right),S_{\text{BC}}\left(r\right)=C_{\text{BC}}\cdot S_{0}\left(r\right)$$

(4) $$\beta \left(r\right)=S_{HC}\left(r\right)/S_{\text{BC}}\left(r\right)=C\left(r\right)/C_{\text{BC}}$$

where S_HC_(r) is the signal acquired with the head coil, C(r) is the receive sensitivity field of the head coil, S_BC_(r) is the signal acquired by the body coil, C_BC_ is the receive sensitivity field of the body coil, assumed to be constant, and S_0_(r) is the signal specific to the underlying anatomy and acquisition parameters. After the sensitivity field has been calculated, the modulation in Eq. (2) can be removed by means of division (as illustrated in Figure 1b):

(5a) $$S_{RB+SC,B}(r,r\prime )=\frac{C\left(r\prime \right)\cdot S_{0}\left(r\right)}{\beta \left(r\prime \right)}=\frac{C\left(r\prime \right)\cdot S_{0}\left(r\right)}{\frac{C\left(r\prime \right)}{C_{BC}}}$$

where S_RB+SC,B_(r,r′) is the detected signal intensity of scan B after receive sensitivity correction and rigid body motion correction. After the division, the receive coil sensitivity modulation is corrected for, and the signal becomes independent of r′.

(5b) $$S_{RB+SC}\left(r,r^{\prime }\right)=S_{0}\left(r\right)\cdot C_{\text{BC}}=S_{RB+SC}\left(r\right)$$

### Estimation of R1 in the Case of Inter‐scan Motion

In the multi‐parameter mapping framework, values of R1 were estimated using a variable flip angle approach. Data from two separate 3D FLASH acquisitions, one predominantly proton density weighted (PDw, flip angle 6°), one predominantly T1 weighted (T1w, flip angle 21°), were used in the estimation, based on rational, small flip angle approximations of the Ernst equation 6, 11:

(6a) $$R1_{\text{RB}}\left(r\right)=\frac{1}{2}\frac{\frac{\left(C(r)\cdot S_{0,B}\left(r\right)\cdot \alpha_{B}(r)\right)}{{\text{TR}}_{B}}\;-\;\frac{\left(C(r)\cdot S_{0,A}\left(r\right)\cdot \alpha_{A}(r)\right)}{{\text{TR}}_{A}}}{\frac{\left(C(r)\cdot S_{0,A}\left(r\right)\right)}{\alpha_{A}(r)}\;-\frac{\left(C(r)\cdot S_{0,B}\left(r\right)\right)}{\alpha_{B}(r)}}$$

(6b) $$R1_{\text{RB}}\left(r\right)=\frac{1}{2}\frac{\frac{\left(S_{0,B}\left(r\right)\cdot \alpha_{B}(r)\right)}{{\text{TR}}_{B}}\;-\;\frac{\left(S_{0,A}\left(r\right)\cdot \alpha_{A}(r)\right)}{{\text{TR}}_{A}}}{\frac{S_{0,A}\left(r\right)}{\alpha_{A}(r)}\;-\;\frac{S_{0,B}\left(r\right)}{\alpha_{B}(r)}}$$

where R1_RB_ denotes the estimated longitudinal relaxation rate after rigid body motion correction, S_0,A_, α_A_ and TR_A_ respectively denote the signal intensity, flip angle and repetition time of the PDw image, and S_0,B_, α_B_, and TR_B_ denote the signal and sequence parameters of the T1w image. The coil receive sensitivity field, C(r), explicitly included in Eq. (6a), is cancelled by division giving Eq. (6b) if there is no motion between the two scans. Local variations in the flip angles, caused by transmit field inhomogeneities, are corrected for using an RF transmit field map 12, 13.

Inter‐scan motion impacts the estimated R1 value even after rigid body registration of the scans, as can be seen from:

(7) $$R1_{\text{RB}}\left(r,r\prime \right)=\frac{1}{2}\frac{\frac{\left(C(r\prime )\cdot S_{0,B}\left(r\right)\cdot \alpha_{B}(r\prime )\right)}{{\text{TR}}_{B}}\;-\;\frac{\left(C(r)\cdot S_{0,A}\left(r\right)\cdot \alpha_{A}(r)\right)}{{\text{TR}}_{A}}}{\frac{\left(C(r)\cdot S_{0,A}\left(r\right)\right)}{\alpha_{A}(r)}\;-\;\frac{\left(C(r\prime )\cdot S_{0,B}\left(r\right)\right)}{\alpha_{B}(r\prime )}}$$

where the position indices r and r′ are analogous to those used in Eq. (2). In this case, the coil receive sensitivity fields, C(r) and C(r′), do not cancel by division, leading to a mismatch between the results of Eqs. [6] and (7). Due to the low spatial variance of the transmit field of the body coil, we assume that for the scale of motion under consideration, α(r′) = α(r).

Applying our proposed correction method by measuring and removing the coil receive sensitivity fields for both positions gives:

(8a) $$S_{RB+SC,A}\left(r\right)=\frac{C\left(r\right)\cdot S_{0,A}\left(r\right)}{\frac{C\left(r\right)}{C_{BC}}}=S_{0,A}\left(r\right)\cdot C_{BC}$$

(8b) $$S_{RB+SC,B}\left(r\right)=\frac{C\left(r\prime \right)\cdot S_{0,B}\left(r\right)}{\frac{C\left(r\prime \right)}{C_{BC}}}=S_{0,B}\left(r\right)\cdot C_{BC}$$

where $S_{RB+SC,A}\left(r\right)$ and $S_{RB+SC,B}\left(r\right)$ are the signal intensity of the PDw and T1w scans after both receive sensitivity field correction and rigid body motion correction. With this correction applied, the scan‐dependent effect of receive sensitivity fields is removed:

(8c) $$R1_{\text{RB}+\text{SC}}\left(r\right)=\frac{1}{2}\frac{\frac{\left(S_{0,B}\left(r\right)\cdot \alpha_{B}\left(r\right)\cdot C_{BC}\right)}{{\text{TR}}_{B}}-\frac{\left(S_{0,A}\left(r\right)\cdot \alpha_{A}\left(r\right)\cdot C_{BC}\right)}{{\text{TR}}_{A}}}{\frac{\left(S_{0,A}\left(r\right)\cdot C_{BC}\right)}{\alpha_{A}\left(r\right)}-\frac{\left(S_{0,B}\left(r\right)\cdot C_{BC}\right)}{\alpha_{B}\left(r\right)}}$$

where R1_RB+SC_ denotes the estimated longitudinal relaxation rate after receive sensitivity correction and rigid body motion correction. The receive sensitivity of the body coil, C_BC_, cancels giving:

(8d) $$R1_{\text{RB}+\text{SC}}\left(r\right)=\frac{1}{2}\frac{\frac{\left(S_{0,B}\left(r\right)\cdot \alpha_{B}\left(r\right)\right)}{{\text{TR}}_{B}}-\frac{\left(S_{0,A}\left(r\right)\cdot \alpha_{A}\left(r\right)\right)}{{\text{TR}}_{A}}}{\frac{S_{0,A}\left(r\right)}{\alpha_{A}\left(r\right)}-\frac{S_{0,B}\left(r\right)}{\alpha_{B}\left(r\right)}}$$

This approach produces an R1 map free of bias induced by inter‐scan motion, as illustrated in Figure 1b.

## METHODS

### Study Design

Five volunteers were instructed to make a single head motion between the acquisitions of two sets of two images with different flip angles, one pair for each of the two positions. This resulted in a total of four structural scans per participant.

Maps of R1 were estimated for each potential pairing of structural scans acquired with the two different flip angles. Thus, four different combinations were used to estimate R1 maps: two with no inter‐scan motion and two with inter‐scan motion.

### Data Acquisition

Data were acquired on a MAGNETOM Trio, a Tim System, 3 Tesla (T) whole‐body MRI system (Siemens Healthcare GmbH, Erlangen, Germany), running software version syngo MR B17. The standard RF body coil was used for transmission. In the case of low‐resolution body coil scans, the body coil was used for reception. In all other cases, a standard 32‐channel RF receive‐only head coil was used.

Two high‐resolution 3D multi‐echo FLASH datasets were acquired in each position, one predominantly proton‐density weighted (PDw, flip angle α=6°) and one predominantly T1 weighted (T1w, α = 21°), leading to four 3D FLASH datasets per volunteer.

Parameters shared by all high‐resolution acquisitions were: field of view (FOV) = 256 × 240 × 176 mm^3^, 1 mm isotropic resolution, repetition time (TR) = 25 ms, first echo time (TE) = 2.34 ms, echo spacing: 2.3 ms, eight echoes, GRAPPA acceleration factor of two in both phase‐encoded directions, with 40 reference lines in each direction, in addition to elliptical k‐space coverage, giving an acquisition time of approximately 4 min per volume.

Two single‐echo, low‐resolution (4 mm isotropic) FLASH scans were acquired before each high‐resolution scan, with identical FOV, and TR/TE/α = 4.64 ms/2 ms/6°. One was acquired receiving on the 32‐channel receive head coil, the other receiving on the body coil. The total acquisition time for the two scans was approximately 25 s.

The local RF transmit field was measured in the first position using spin and stimulated echoes acquired using a 3D EPI sequence, with the following parameters: FOV = 256 × 192 × 192 mm^3^, 4 mm isotropic resolution, TR/TE/mixing time: 500/37.06/31.2 ms. Eleven nominal flip angles were used ranging from 65° to 115° in steps of 5°. An additional B_0_ field map was acquired to account for distortions in the EPI readout 12, 13.

### Participants

Five healthy participants (age range: 33–43 years, 2 males) were scanned for this study, approved by the local Ethics committee. Written informed consent was obtained from all participants.

### Participant Motion

Volunteers were instructed to make a single head motion between the acquisitions of the two sets of images. They were asked to move between 10 and 20 mm in one continuous motion that included a nod, in a direction out of the bore (toward the feet). The extent of motion was chosen to be at the higher end of the range reported in patients 14, or used to evaluate intrascan motion correction methods 15, 16 to robustly assess the method. The extent of motion was estimated retrospectively using rigid body registration, as implemented in SPM12b (http://www.fil.ion.ucl.ac.uk/spm/). To control for undesired motion between the two scans acquired at the same position, their relative position was also estimated using rigid body registration. The means and standard deviations (SD) of the amplitudes of the six motion parameters across all volunteers were calculated.

### Image Processing

Data were processed using SPM12b and custom‐made scripts in MATLAB 7.14 (The Mathworks, Natick, MA). Four maps of the apparent relaxation rate were estimated per participant, one estimated from data within the first position (first identical position case), one estimated from data within the second position (second identical position case), one estimated from the PDw scan in the first and the T1w scan in the second position (first inter‐scan motion case), one estimated from the T1w scan in the first and the PDw scan in the second position (second inter‐scan motion case). These R1 maps were calculated with and without inter‐scan receive sensitivity field correction, giving a total of eight maps per participant.

The correction of inter‐scan motion artifacts related to coil receive sensitivity changes required reliable coil receive sensitivity maps for each high‐resolution scan, estimated from the two low‐resolution images after coregistration to the corresponding high‐resolution structural scan 17, including resampling to the higher resolution of 1 mm^3^. These higher‐resolution calibration images were then smoothed with a Gaussian smoothing kernel with a full width at half maximum of 12 mm. This kernel width was selected to correct for artifacts arising from the resampling and co‐registration processes, while preserving the spatial details of the combined coil sensitivity. After smoothing, the image acquired with the 32‐channel RF head coil was voxel‐wise divided by the image acquired with the RF body coil (as in Eq. (4)). This resulted in an image of the combined spatial receive sensitivity field of the 32‐channel head coil. This net modulation was removed from the first six echoes of the high‐resolution PDw and T1w acquisitions by voxel‐wise division (as in Eqs. (5a), (5b)).

Subsequent processing steps were identical for all data. In brief, the arithmetic means of the first six echoes for all high‐resolution scans were calculated to increase the signal to noise ratio 18. Maps of the apparent R1 were calculated according to the previously published method 6. This includes rigid body motion correction to align the PDw and T1w acquisitions, and correction of RF transmit field inhomogeneities 12, 13. This resulted in four maps with rigid body motion correction (R1_RB_), and four with rigid body motion correction and coil receive sensitivity correction (R1_RB+SC_) per participant.

All eight maps were co‐registered to the R1_RB_ map estimated from data acquired in the first position, termed R1_1_ below. Tissue probability maps were estimated from both the R1_RB_ and the R1_RB+SC_ maps estimated from data acquired in the first position using the unified segmentation algorithm implemented in SPM12b 19. The resulting two gray matter probability maps were tresholded at 95% probability, and their conjunction was used as a tissue specific mask for gray matter. A tissue specific mask for white matter was derived in a similar manner. Probability maps from both correction methods were used to account for residual image processing artifacts and to minimize bias toward a particular method.

The different R1 maps were compared with R1_1_ by calculating the normalized mean root square error (MRSE) of the difference between the R1 map of interest and R1_1_ for all voxels within the gray and white matter tissue masks:

(9) $$\text{MRSE}=\frac{1}{N}\underset{j=1:N}{\sum }\sqrt{{\left(\frac{R1_{i}\left(j\right)\;-\;R1_{1}(j)}{R1_{1}(j)}\right)}^{2}}\cdot 100\%$$

where N was the number of voxels in the tissue masks, R1_1_(j) is the value of voxel j of R1_1_, and R1_i_(j) is the value of a different R1 map at voxel j.

To assess how much the motion artifacts affected the homogeneity of the R1 maps, a coefficient of variation (CoV), defined as the standard deviation over the mean, was calculated across all the voxels within each tissue type for all maps and all participants. The tissue masks were large regions of interest, but covering rather homogeneous tissue. A conjunction of all probability maps (both gray matter and both white matter probability maps), with a probability threshold of >35% was used to mask R1 maps for visual inspection, and to generate voxel‐wise difference maps.

## RESULTS

The participants executed head motion as instructed. The range of motion between the two positions was approximately ten times larger than the range of undesired motion within positions. Figure 2 shows the translation and rotation parameters across the group.

**Figure 2:**
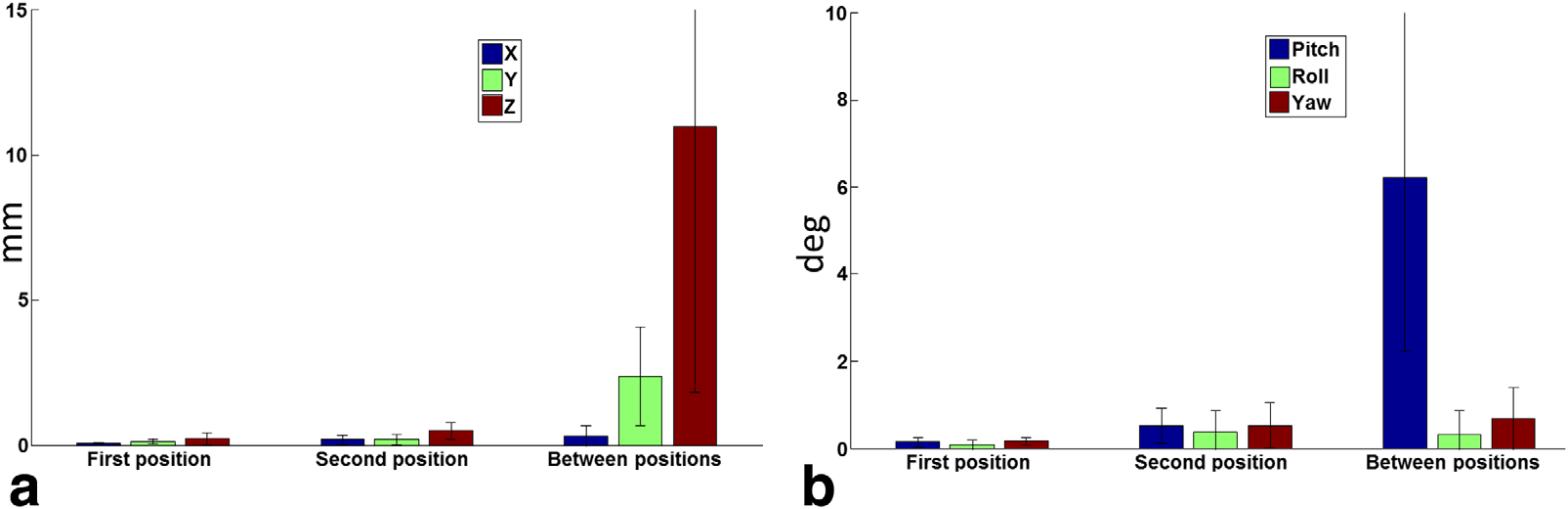
Translation (**a**) and rotation (**b**) parameters (mean ± SD across all participants) for within (undesired) and between (instructed) position motion.

The MRSE of the eight maps with respect to R1_1_ are summarized in Table 1. The MRSE values for the two inter‐scan motion cases were twice (∼16%) the MRSE for the R1_RB_ maps for the second identical position case (∼7.5%). As no instructed motion occurred, the second identical position case is effectively a case without inter‐scan motion. For R1_RB+SC_ maps, MRSE for the two inter‐scan motion cases were comparable with the MRSE for the second identical position case. In other words, the inter‐scan motion induced error was reduced to the unavoidable scan‐rescan error.

**Table 1:** Mean Root Square Error (MRSE)^a^

Motion case	Correction method	
	Rigid body	Rigid body with sensitivity correction
First identical position case		1.94 ± 0.48%
Second identical position case	7.54 ± 1.39%	7.31 ± 1.41%
First inter‐scan motion case	14.79 ± 5.33%	6.10 ± 1.78%
Second inter‐scan motion case	17.45 ± 9.73%	6.18 ± 0.76%

a MRSE was measured against R1_1_, the R1_RB_ map of the first identical‐position case for all estimations of R1 using both correction methods (mean±SD across the group). MRSE for rigid body corrected interscan motion cases was twice the scan‐rescan variability (defined as the MRSE of the second identical motion case). Additional receive sensitivity correction reduced MRSE below the level of scan**–**rescan variability.

The CoV within gray and white matter masks (mean ± SD across the group) are summarized in Table 2. For both motion cases, CoV in R1_RB_ maps was increased compared with the identical position cases. In R1_RB+SC_ maps, the CoV was comparable for the inter‐scan motion cases and identical position cases.

**Table 2:** Coefficient of Variation (CoV)^a^

Motion case	Tissue mask	Correction method	
		Rigid body	Rigid body with sensitivity correction
First identical position	Gray matter	0.122 ± 0.006	0.124 ± 0.004
	White matter	0.083 ± 0.009	0.084 ± 0.009
Second identical position	Gray matter	0.144 ± 0.018	0.137 ± 0.017
	White matter	0.088 ± 0.013	0.083 ± 0.011
First inter‐scan motion	Gray matter	0.206 ± 0.035	0.136 ± 0.010
	White matter	0.130 ± 0.027	0.089 ± 0.010
Second inter‐scan motion	Gray matter	0.195 ± 0.037	0.138 ± 0.018
	White matter	0.140 ± 0.026	0.087 ± 0.018

a Both correction methods were compared for both tissue types (mean±SD across all participants). CoV was greatly increased for inter‐scan motion cases with only rigid body motion correction, while additional receive sensitivity correction resulted in a CoV comparable to the first identical position case.

Typical sensitivity maps are shown in Figure 3. Here, the sensitivity maps for the PDw scans in both positions were calculated and co‐registered for volunteer 2.

**Figure 3:**
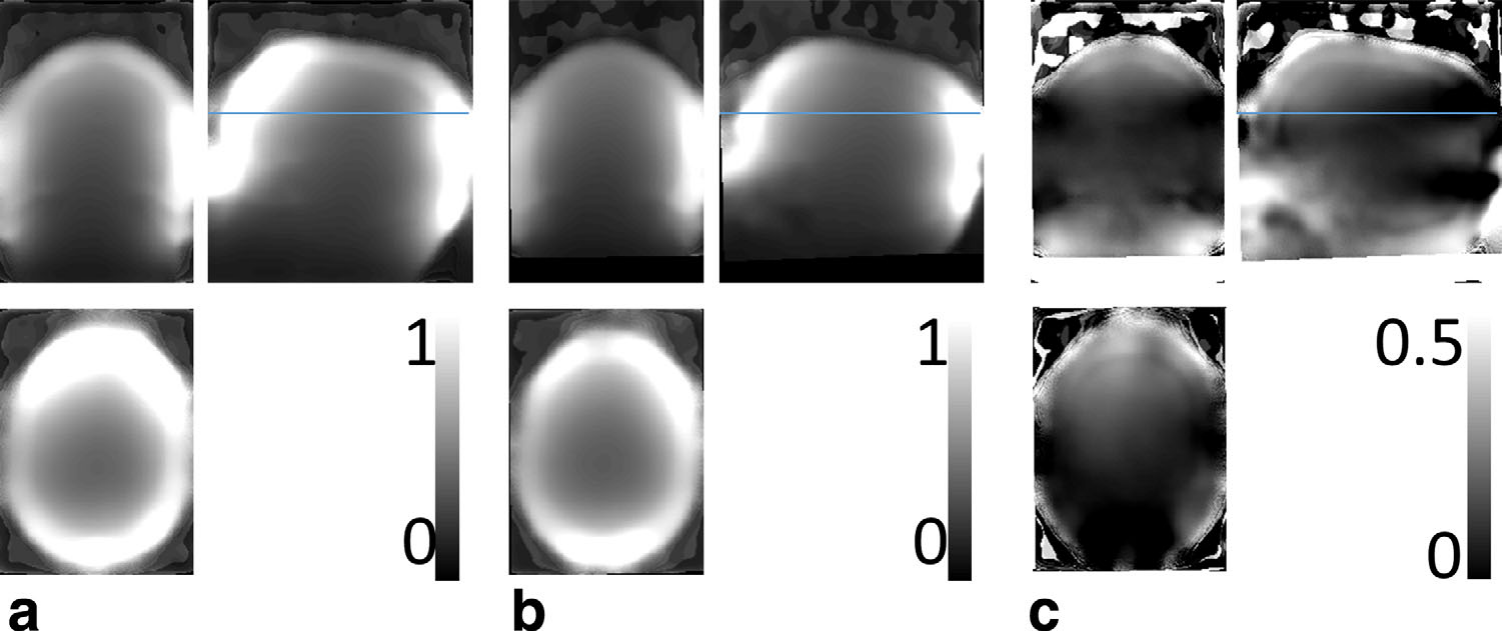
RF receive sensitivity maps for the PDw acquisitions of participant 2: measured for the first position (**a**), the second position (**b**), and their difference (**c**). The sensitivity was high at the periphery and dropped off toward the center of the brain. The difference of the two fields (c) reflected the sensitivity change resulting from inter‐scan motion and showed the largest differences in the periphery of the brain. The line indicates the position of the two difference maps shown in Figures 4e,f and g,h.

**Figure 4:**
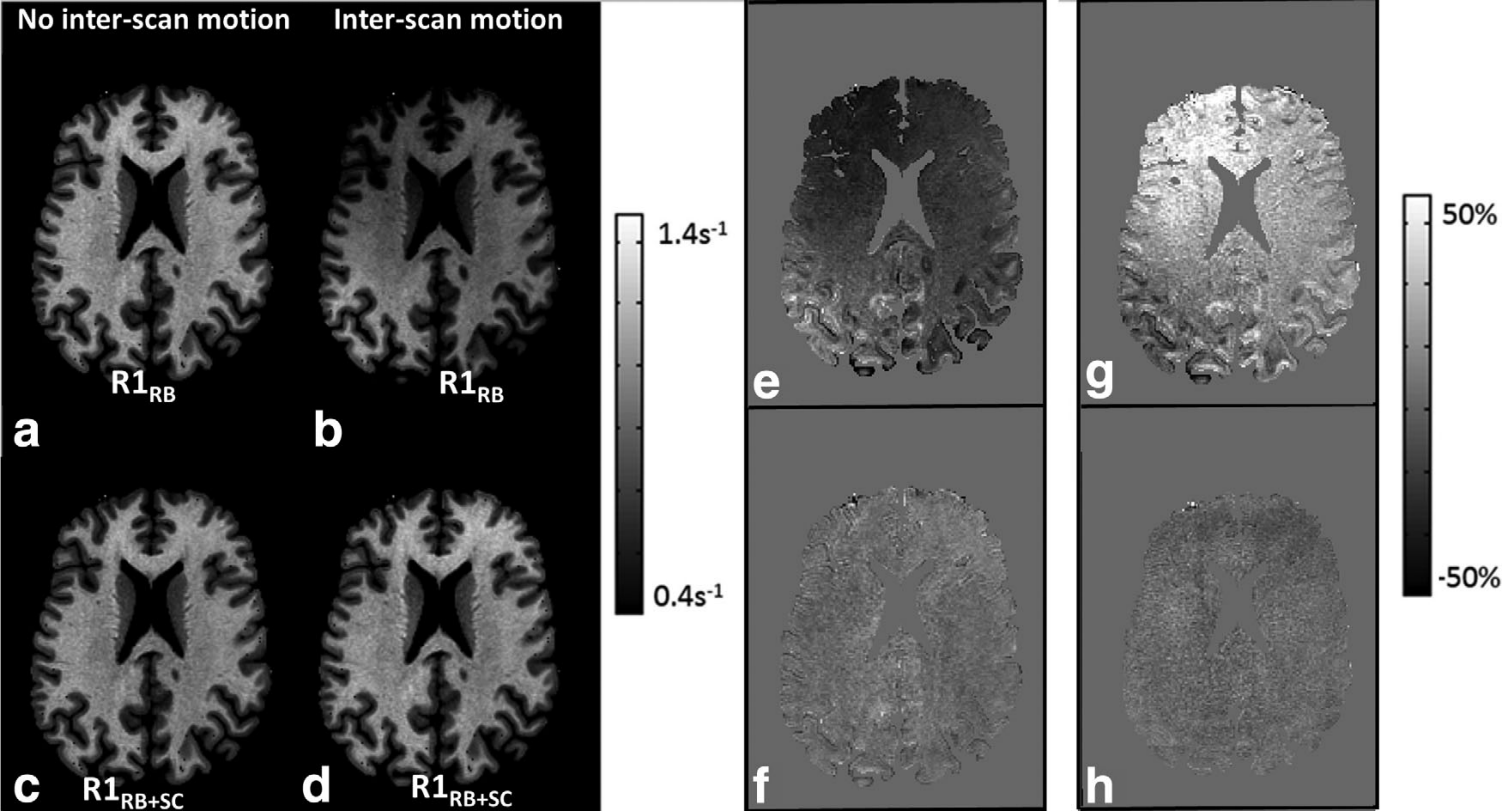
R1 maps for participant 2 corrected with rigid body motion correction or rigid body motion correction and additional receive sensitivity correction. **a**: First identical position case, R1_RB_. **b**: First motion case, R1_RB_. **c**: First identical‐position case, R1_RB+SC_. **d**: First motion case, R1_RB+SC_. **e**: Difference between (a) and (b). **f**: Difference between (c) and (d). The bias introduced by inter‐scan motion is mainly apparent as an anterior–posterior gradient (e) that was removed by the sensitivity correction (f). Difference maps for the second interscan motion case are also shown: difference map for R1_RB_ maps of the second interscan motion case (**g**); difference map for the R1_RB+SC_ maps of the second interscan motion case (**h**).

The impact of inter‐scan motion on image quality is shown in Figure 4. Inter‐scan motion resulted in an anterior–posterior gradient in the R1_RB_ map (Fig. 4b), particularly evident on the difference map (Fig. 4e), even after rigid body motion correction. Additional receive sensitivity correction reduced this artifact to a negligible level (Figures 4d and f, compare Figures 4g and h).

## DISCUSSION

Inter‐scan motion between different scans acquired for quantitative R1 mapping resulted in prominent artifacts in the calculated R1 maps. These artifacts are not corrected by conventional rigid body motion correction. We introduced a method that additionally accounts for variations in RF receive sensitivity fields caused by relative motion of the head with respect to the RF receive coils between scans. This correction scheme reduced the inter‐scan motion artifact level to that typical of scan–rescan variability. The overall image quality was significantly improved and spurious motion‐related spatial gradients in the R1 maps were removed.

### Impact of Inter‐scan Motion

Inter‐scan motion introduced bias into the R1 maps that was greater than the scan–rescan variability that was determined by comparing the two datasets unaffected by inter‐scan motion. The MRSE for the scan–rescan experiment was more than doubled, with the mean value for the group rising from ∼7.5% to ∼16%.

The R1 maps were severely biased by inter‐scan motion and showed reduced homogeneity, an effect that is visually discernable. Inter‐scan motion increased the CoV in gray matter by ca. 64% and in white matter by ca. 62%.

The bias and artifacts introduced by inter‐scan motion originate from changes in the RF receive sensitivity fields due to the altered positioning of the head within the receive coil. The largest motion‐related biases in the R1 maps were observed in superficial cortical brain areas, i.e. in areas with steep sensitivity gradients (e.g., frontal cortex, compare Figures 3c and 4), while significant biases are observable through the brain. The rapidly varying spatial sensitivity of the receive coil means that even rather small head movements can lead to appreciable signal changes and therefore bias in the calculated R1 value, particularly when the movement occurs in areas close to the receive coils.

### Correction for Inter‐scan Motion

For both motion cases, MRSE of the R1_RB+SC_ maps was reduced below the level of inter‐scan variability, to ∼6% for both inter‐scan motion cases, as compared to the inter‐scan variability of ∼7.5% determined without the additional receive sensitivity correction (which is therefore also affected by small inter‐scan motion effects). The MRSE of the R1_RB+SC_ map for the first identical position case is ∼2%. This may indicate that our proposed correction method additionally corrects for small, unintentional inter‐scan motion within the first position, hence, the observed differences between the R1_RB_ and R1_RB+SC_ maps estimated in the first identical position case.

With additional receive sensitivity correction, increases in CoV for both inter‐scan motion cases were reduced from ∼64% to ∼12% in gray, and from ∼62% to ∼6% in white matter, comparable to the difference caused by scan–rescan variability.

Additional receive sensitivity correction did not change the visual image quality for the corrected first position case (as seen in Figures 4c and 4a). This is to be expected since the data were minimally affected by inter‐scan motion as evidenced by the realignment parameters. Our method corrected the visually apparent bias caused by inter‐scan motion (as seen in Figures 4d and 4b).

### Potential Issues and Applications

The proposed correction method addresses inter‐scan motion but not intra‐scan motion, which may affect both the two short scans that are used to calculate the receive sensitivity field, as well as the high‐resolution structural scans. To address the effects of intra‐scan motion, the proposed sensitivity correction could be combined with advanced motion correction methods, such as prospective motion correction based on optical tracking 20.

Receive sensitivity fields are estimated based on the assumption that the receive sensitivity field of the body coil is flat compared to the receive sensitivity field of the multichannel head coil. Deviations from this assumption will lead to residual errors that are not corrected by the method. However, as the receive sensitivity field of the body coil can always be assumed to be flatter than the head coil, the proposed correction method will always lead to an improvement.

Our proposed method does not address the effect of inter‐scan motion on the transmit field. To evaluate the impact of inter‐scan motion on the transmit field, B_1_^+^ maps were acquired in the second position at the end of the scanning session for three of the five participants. For these participants, the B_1_^+^maps from the two positions were co‐registered, and masked in the same manner as the R1 maps. The MRSE of the second‐position B_1_^+^ maps was (mean ± SD over the three participants) 1.41 ± 0.61%. In our method for estimating R1, errors in the estimation of the transmit field translate to errors in the R1 map in a quadratic manner 6, thus the impact of these errors, approximately 2.3%, is an order of magnitude lower than the MRSE of the two inter‐scan motion cases. In addition, the distribution of voxel‐wise MRSE values for the B_1_^+^ maps were found to be unimodal. This indicates that changes in the transmit field alone are not the main source of the error observed in R1 maps.

The proposed method was demonstrated in a VFA approach to measuring R1 but could be expanded to all quantitative MRI methods that rely on the combination of multiple scans acquired consecutively. The short scan time (totaling ∼25s for the two low‐resolution scans) required by the proposed method would have little impact on the duration of typical protocols.

## CONCLUSIONS

Quantitative R1 mapping is significantly affected by signal changes caused by movement of the head through the RF coil receive sensitivity field. This effect is not corrected for by conventional rigid body motion correction. Our proposed method, based on the estimation of receive sensitivities individually for each constituent scan, reduces the impact of inter‐scan motion to a level comparable with scan–rescan variability, while preserving visual image quality. This novel method has been demonstrated for the VFA approach to R1 mapping but is applicable to all quantitative MRI methods that rely on combining sequentially acquired scans.
